# Meta‐analysis of microarray data to determine gene indicators involved in the cisplatin resistance in ovarian cancer

**DOI:** 10.1002/cnr2.1884

**Published:** 2023-11-07

**Authors:** Somayeh Hashemi Sheikhshabani, Zeinab Amini‐Farsani, Nesa Kazemifard, Parastoo Modarres, Zahra Amini‐Farsani, Mir Davood Omrani, Soudeh Ghafouri‐Fard

**Affiliations:** ^1^ Student Research Committee, Department of Medical Genetics Shahid Beheshti University of Medical Sciences Tehran Iran; ^2^ Department of Medical Genetics Shahid Beheshti University of Medical Sciences Tehran Iran; ^3^ Basic and Molecular Epidemiology of Gastrointestinal Disorders Research Center, Research Institute for Gastroenterology and Liver Diseases Shahid Beheshti University of Medical Sciences Tehran Iran; ^4^ Department of Cell and Molecular Biology and Microbiology University of Isfahan Isfahan Iran; ^5^ Bayesian Imaging and Spatial Statistics Group, Institute for Statistics Ludwig‐Maximilians‐Universität München Munich Germany; ^6^ Statistics Department School of Science, Lorestan University Khorramabad Iran; ^7^ Urogenital Stem Cell Research Center Shahid Beheshti University of Medical Sciences Tehran Iran

**Keywords:** cisplatin resistance, meta‐analysis, ovarian cancer

## Abstract

**Background:**

Significant miss‐expressed gene indicators contributing to cisplatin resistance in ovarian cancer have not been completely understood. It seems that several regulatory genes and signaling pathways are associated with the emergence of the chemo‐resistant phenotype.

**Aims:**

Here, a meta‐analysis approach was adopted to assess deregulated genes involved in relapse after the first line of chemotherapy (cisplatin).

**Methods and Results:**

To do so, six ovarian cancer libraries were gathered from GEO repository. Batch effect removal and quality assessment, and boxplots and PCA were performed using SVA and ggplot2 packages in R, respectively. Cisplatin‐resistant and ‐sensitive ovarian cancer groups were compared with find genes with significant expression changes using linear regression models in the LIMMA R package. The significance threshold for DEGs was taken as adj *p*‐value < .05 and − 1 > logFC > 1. A total of 261 genes were identified to have significant differential expression levels in the cisplatin‐resistant versus cisplatin‐sensitive group. Among the 10 top up‐regulated and down‐regulated genes, *PITX2*, *SNCA*, and *EPHA7* (up), as well as *TMEM98* (down) are indirect upstream regulators of PI3K/AKT signaling pathway, contributing greatly to the development of chemo‐resistance in cancer via promoting cell proliferation, survival, and cell cycle progression as well as inhibiting apoptosis. Moreover, a comprehensive assessment of DEGs revealed the dysregulation of not only membrane ion channels KCa1.1, Kv4, and CACNB4, affecting cell excitability, proliferation, and apoptosis but also cell adhesion proteins COL4A6, EPHA3, and CD9, affecting the attachment of normal cells to ECM and apoptosis, introducing good options to reverse cisplatin resistance.

**Conclusion:**

Our results predict and suggest that upstream regulators of PI3K/AKT signaling pathway, ion channels, and cell adhesion proteins play important roles in cisplatin resistance development in ovarian cancer.

## 1 INTRODUCTION

Ovarian cancer (OC) is ranked as the ninth most prevalent malignancy worldwide among the female population and the eighth major cause of cancer‐related mortality. In 2022, around 19 880 new OC cases and 12 810 related deaths were reported in the United States alone.^1^ Epithelial ovarian carcinoma (EOC) has the highest frequency and comprises 90%–98% of ovarian tumors.^2^ EOC is often referred to as the “silent killer” since it demonstrates a few symptoms until its metastasis within the peritoneal cavity, which corresponds to a significantly reduced chance of curing.^3^

The usual treatment procedure for OC patients includes cytoreductive surgery and then chemotherapy with paclitaxel, carboplatin, or cisplatin. Nevertheless, ~70% of patients receiving this treatment will experience tumor relapse.^4^, ^5^ This necessitates understanding the molecular mechanisms of platinum resistance. Cisplatin serves as a highly effective chemotherapy drug for the treatment of a wide variety of cancers such as OC. Cisplatin targets cancerous cells by forming adducts/crosslinks with DNA purine bases, preferably with guanine. These crosslinks damage DNA; this in turn suppresses proper gene replication and transcription and induces cell apoptosis.^6^ Despite proper early response rate, the majority of patients treated with cisplatin will suffer later relapse after 6 months. Tumor recurrence more than 6 months following front‐line platinum treatment is known as sensitivity to platinum, while recurrence before 6 months is regarded as resistance. Progression of OC during the 6‐month period after the end of platinum‐based chemotherapy is normally related to platinum resistance.^7^ Due to its significant impact on patient survival time and quality, improving the response to cisplatin through discovering key genes and signaling pathways involved in cisplatin resistance is an important subject and paves the way for cancer treatment in the future.

According to the literature, resistance to platinum‐based drugs is associated with a number of cellular events including decreased concentration of the platinum compounds by either active efflux/sequestration/secretion or impaired influx, detoxification by GSH conjugates, high DNA damage repair levels (nucleotide excision repair and mismatch repair), changed DNA methylation status, altered membrane protein trafficking due to faulty distribution and organization of the cytoskeleton, overexpression of chaperones, modification of microRNA (miRNA), long non‐coding RNA (lncRNA) expression, transcription factors, and small GTPases, inactivated apoptosis pathway, and activated EMT pathway.^7^, ^8^, ^9^ Nevertheless, insufficient information regarding molecular mechanisms involved in the cisplatin‐resistance in OC prevents the development of efficient targeted therapy for eliminating cancer cells.^10^ Hence, identifying new target genes is of particular interest in terms of discovering molecular mechanisms involved in the cisplatin resistance in OC and finally obtaining molecular biomarkers capable of indicating pharmacological response in the body and novel efficient treatment strategies for patients with advanced OC.^11^

There has been increasing interest in the use of integrated analysis for examining multiple independent microarray data sets.^12^ Based on increasing findings, meta‐analysis enhances the statistical power of expression profiling and allows for an investigation of cross‐study heterogeneity; this can result in more reliable and robust gene signatures.^13^ Hence, in this work, a meta‐analysis was carried out on multiple public microarray‐based data sets regarding chemotherapeutic response in OC to discover strong gene expression signatures and pathways related to the cisplatin‐resistance.

## 2 METHODS

### 2.1 Data set selection approach

**FIGURE 1:**
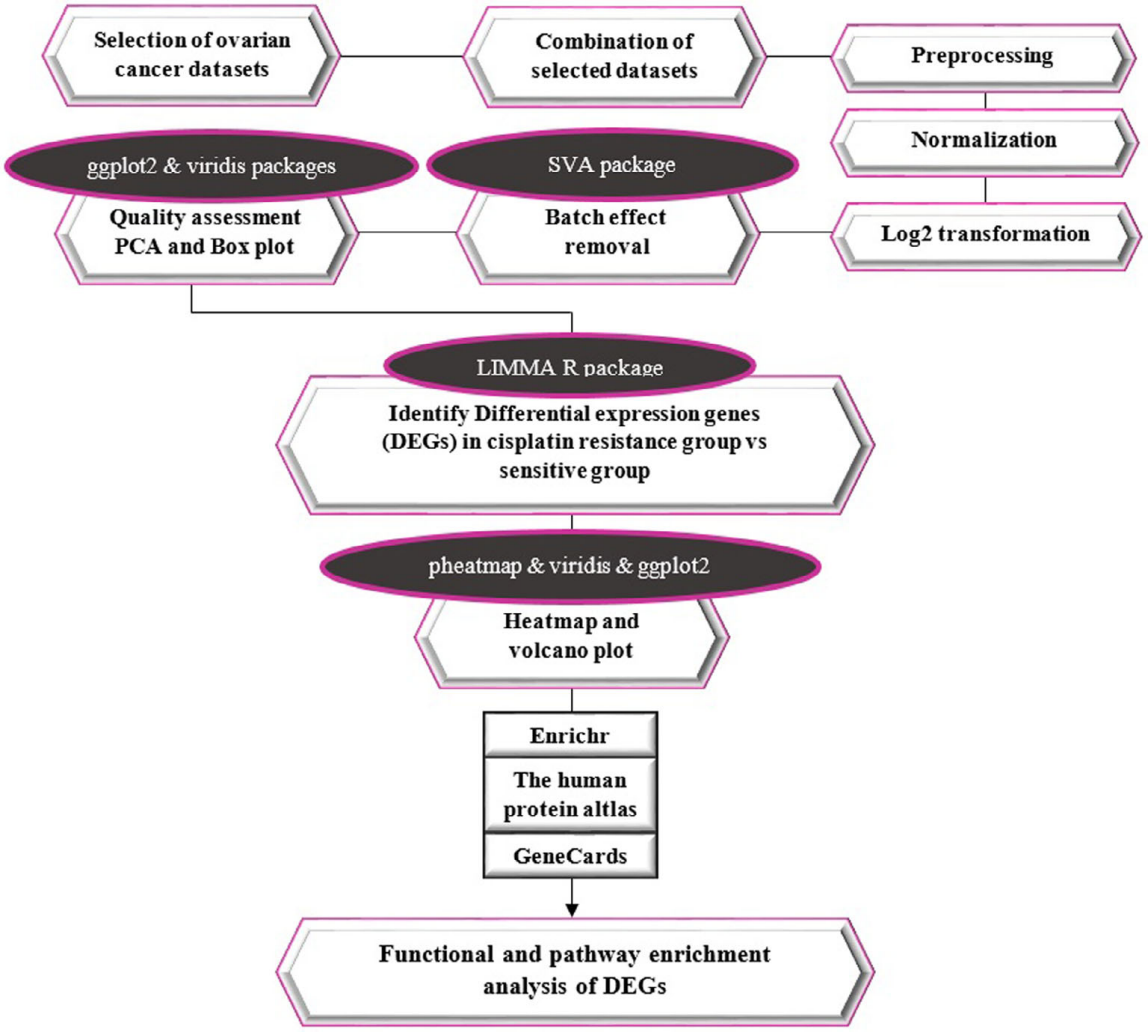
Meta‐analysis workflow of microarray data sets.

Gene Expression Omnibus (GEO) (https://www.ncbi.nlm.nih.gov/gds) repository was explored, and corresponding ovarian drug resistance published array expression data sets were retrieved. The MeSH terms searched for the current study included “Ovarian neoplasms, Carcinoma/Ovarian Epithelial” and “Drug resistance, Recurrence” and “Cisplatin.” In order to limit the results, two filters, namely “Homo sapiens” and “Expression profiling by array” were selected. Then, inconsistent data sets and duplicates were removed by including several criteria, such as cells (epithelial) or OC patients, treatment (a minimum of one chemotherapy session using cisplatin), and platforms of microarray tests. Afterward, six microarray data sets were selected with two different platforms, namely Affymetrix and Agilent single‐channel arrays. Furthermore, exclusion criteria for studies included the unavailable raw data and low quality. Here, the considered chemotherapy regimen consisted of only cisplatin. Thus, data sets that included cisplatin regimen combined with other anti‐cancer drugs with DNA‐damaging effects including carboplatin, taxol, or doxorubicin were removed. According to the above‐mentioned criteria, samples were arranged in two distinct groups: cisplatin‐sensitive (total disappearance after the first treatment) and cisplatin‐resistance (recurrence). The results considered here were derived from epithelial OC cell lines as well as OC patients' samples. All data sets of cell lines were combined and analyzed together and patient data sets were analyzed separately. In addition, only the A2780 cell line was selected for further analysis. For each case, a different cross‐integration analysis was carried out, and gene set enrichment analysis (GSEA) was conducted for common differentially expressed genes (DEGs). Figure 1 shows the general research workflow.

### 2.2 Data collection and filtering

This analysis was carried out on five data sets GSE73935 (GPL13667), GSE51683 (GPL6244), GSE23553 (GPL570), GSE15372 (GPL570), and GSE131978 (GLP96, GPL570) with Affymetrix platforms and one data set, GSE33482 with Agilent‐014850 (GPL6480) platform. These data sets were as follows: (1) GSE73935, which included the gene expression profiles of nine biological replica of cisplatin resistance OC cell line, A2780CR. (2) GSE51683, which involved the gene expression profiles of human OC cell lines A2780 and A2780CP20. Profiles included two data sets that were sensitive and two data sets that were resistant to cisplatin. Four expression profiles were excluded from this work due to being treated with temsirolimus; (3) GSE23553, which included RNA expression profiles of one cisplatin‐sensitive, A2780S, and one cisplatin‐resistant A2780CP cell line. (4) GSE15372, which consisted of the gene expression profiles of five biological copies of A2780 cell line (cisplatin‐sensitive) together with round5 A2780 cell line (cisplatin‐resistant), the latter obtained by culturing A2780 cells and exposing them to several cycles (treatment rounds) with incremental cisplatin doses. (5) GSE33482, which involved the expression profiles of six biological copies of A2780 (cisplatin‐sensitive) together with six biological copies of A2780cis (cisplatin‐resistant) OC cell line; and (6) GSE131978, which comprised of the expression profiles of 39 OC patients who had received cisplatin as a chemotherapy drug. These profiles included 16 samples that were sensitive and 7 samples that were resistant to cisplatin. Sixteen expression profiles were excluded from this work due to being unclear.

### 2.3 Normalization of raw data and quality control

Preprocessing, quality control, and human GRCh38 alignment were carried out for all data sets, and raw read counts were downloaded for additional study. Removal of potential background noise, non‐specific binding, and log2 transformation were conducted for each selected data set. In the following, batch effect removal and data normalization after data integration were performed using surrogate variable analysis (SVA) package. The normalization quality and data distribution were assessed by drawing the pre‐ and post‐normalization boxplots. Other quality assessment approaches included the pre‐ and post‐normalization evaluation and bath effect removal between array data sets by principal component analysis (PCA) using ggplot2 package in R.

### 2.4 Integrative meta‐analysis

PCA plots were developed on the integrated data to examine the level of batch effect (or undesirable variation) on the data. A comparison was made between OC groups resistant to cisplatin and those sensitive to it, and differentially expressed genes were identified with no regard to French‐American‐British (FAB) classification, drug dose, and sample source. Moreover, another meta‐analysis was carried out with arrays of the samples of patients (cisplatin‐resistant and cisplatin‐sensitive patients) to obtain a more precise set of DEGs participating in the cisplatin‐resistance in OC.

### 2.5 Gene set enrichment analysis

Gene Ontology (GO) and pathway enrichment analysis were performed for DEGs through the web‐based enrichment analysis tool, Enrichr (https://maayanlab.cloud/Enrichr/), containing >180 000 curated gene sets in multiple categories from >100 gene set libraries. Moreover, the human protein atlas (https://www.proteinatlas.org) and GeneCards (https://www.genecards.org) were used for further investigation of DEGs. To better illustrate the key DEGs that contributed to OC drug resistance, heatmap graph was pictured using pheatmap and viridis packages in R.

### 2.6 Statistical analysis

The R statistical computer program was used to perform all statistical analyses. Linear regression models in linear models for microarray data (LIMMA) R program were used to analyze differential gene expression obtained from comparing cisplatin‐resistant cell line to cisplatin sensitive cell line. The significance thresholds for DEGs were taken as adj *p*‐value < .05 and logFC > 1 or log FC < −1. In addition, via the Benjamini–Hochberg (B–H) method, the t‐statistics test results were analyzed and false positive results were reduced.

## 3 RESULTS

### 3.1 Data collection and filtering

We matched six publicly available microarray data sets comprising a total of 57 arrays to our pre‐specified criteria for inclusion. Table 1 gives the details of the aforementioned six data sets. By eliminating outliers and inconsistent sample arrays, data sets were normalized with 57 sample arrays, which were considered for more downstream analyses. Afterward, we divided the samples into two subgroups when conducting the meta‐analysis: chemo‐sensitive and chemo‐resistant. Table 2 shows brief information of cell line samples used in this meta‐analysis.

**TABLE 1:** Characteristics of the gene expression data sets applied in the meta‐analysis.

Accession no	Platform	Drugs	Tissue	Sample	Number of samples		
					Selected/total	Sensitive	Resistance
GSE73935	GPL13667, affymetrix	Cisplatin, doxorubicin, topotecan, paclitaxel	Ovarian cancer	A2780 cell line	6/48	‐	6
GSE51683	GPL6244, affymetrix	Cisplatin, temsirolimus	Ovarian cancer	A2780 cell line	4/8	2	2
GSE23553	GPL570, affymetrix	Cisplatin	Ovarian cancer		2/56	1	1
GSE15372	GPL570, affymetrix	Cisplatin	Ovarian cancer	A2780 cell line	10/10	5	5
GSE33482	GPL6480, agilent	Cisplatin	Ovarian cancer	A2780 cell line	12/12	6	6
GSE131978	GPL96, GPL570 affymetrix	Cisplatin	Ovarian cancer	Patients	23/39	16	7

**TABLE 2:** Characteristics of the samples applied in the meta‐analysis and further investigation.

Accession	Group	Study	Platform
GSM1906460	Resistant	GSE73935	HG‐U219
GSM1906461	Resistant	GSE73935	HG‐U219
GSM1906462	Resistant	GSE73935	HG‐U219
GSM1906463	Resistant	GSE73935	HG‐U219
GSM1906464	Resistant	GSE73935	HG‐U219
GSM1906465	Resistant	GSE73935	HG‐U219
GSM1250444	Sensitive	GSE51683	HuGene‐1_0‐st
GSM1250445	Sensitive	GSE51683	HuGene‐1_0‐st
GSM1250446	Resistant	GSE51683	HuGene‐1_0‐st
GSM1250447	Resistant	GSE51683	HuGene‐1_0‐st
GSM577774	Resistant	GSE23553	HG‐U133_Plus_2
GSM577781	Sensitive	GSE23553	HG‐U133_Plus_2
GSM385721	Sensitive	GSE15372	HG‐U133_Plus_2
GSM385722	Sensitive	GSE15372	HG‐U133_Plus_2
GSM385723	Sensitive	GSE15372	HG‐U133_Plus_2
GSM385724	Sensitive	GSE15372	HG‐U133_Plus_2
GSM385725	Sensitive	GSE15372	HG‐U133_Plus_2
GSM385726	Resistant	GSE15372	HG‐U133_Plus_2
GSM385727	Resistant	GSE15372	HG‐U133_Plus_2
GSM385728	Resistant	GSE15372	HG‐U133_Plus_2
GSM385729	Resistant	GSE15372	HG‐U133_Plus_2
GSM385730	Resistant	GSE15372	HG‐U133_Plus_2
GSM828379	Sensitive	GSE33482	Agilent
GSM828380	Sensitive	GSE33482	Agilent
GSM828381	Sensitive	GSE33482	Agilent
GSM828382	Sensitive	GSE33482	Agilent
GSM828383	Sensitive	GSE33482	Agilent
GSM828384	Sensitive	GSE33482	Agilent
GSM828385	Resistant	GSE33482	Agilent
GSM828386	Resistant	GSE33482	Agilent
GSM828387	Resistant	GSE33482	Agilent
GSM828388	Resistant	GSE33482	Agilent
GSM828389	Resistant	GSE33482	Agilent
GSM828390	Resistant	GSE33482	Agilent

### 3.2 Quality evaluation of calibrated data

**FIGURE 2:**
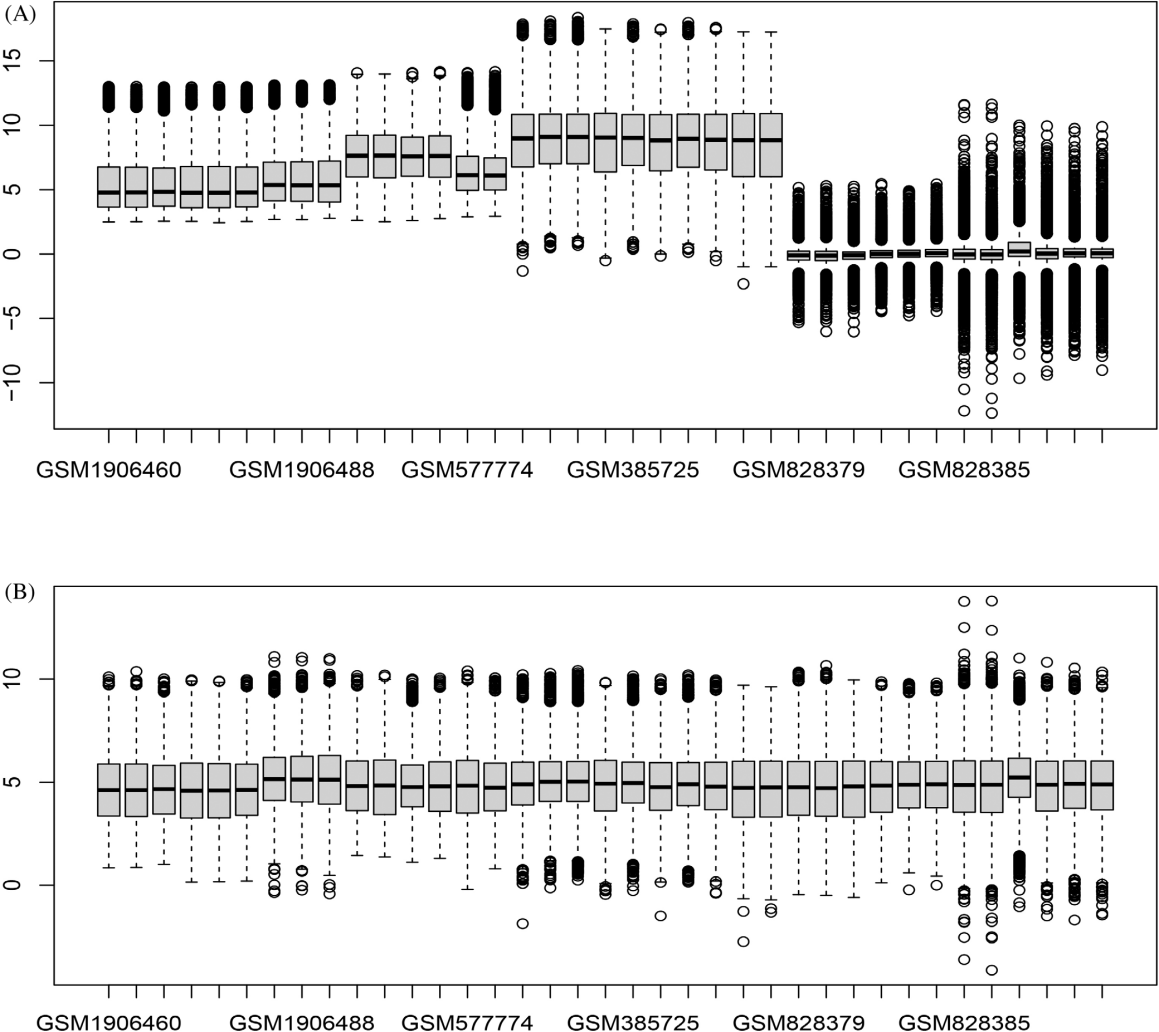
Boxplot of ovarian cancer sample arrays (A) before and (B) after batch correction. The concentrated distribution of chips around zero indicates that undesired variation has almost been eliminated. R language program was used to develop the plot.

**FIGURE 3:**
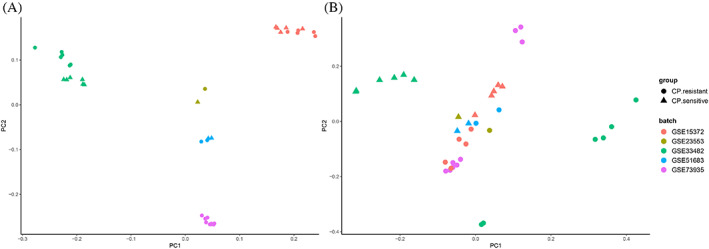
PCA plots of data summarized via batch correction. PCA scatter plots give PC1 versus PC2 outputs for each calibrated ovarian cancer sample of data sets (A) before and (B) after batch correction. PCA plots developed using ggplot2 package version 3.3.3 in R indicate that gene expression profiles of samples are similar.

The batch effect was reduced by applying the LIMMA, which is a well‐established procedure (Figure 2). As can be seen in Figure 2, in most cases, chips are mainly distributed around zero. Our analysis showed no significant differences representing a bias. Furthermore, the clustering patterns of the sample were assessed by presenting the results by PCA plots. This plot shows clustering primarily according to cisplatin‐resistant and ‐sensitive series (Figure 3).

### 3.3 Statistical meta‐analysis

Identifying DEG signatures in chemo‐resistant OC samples.

**FIGURE 4:**
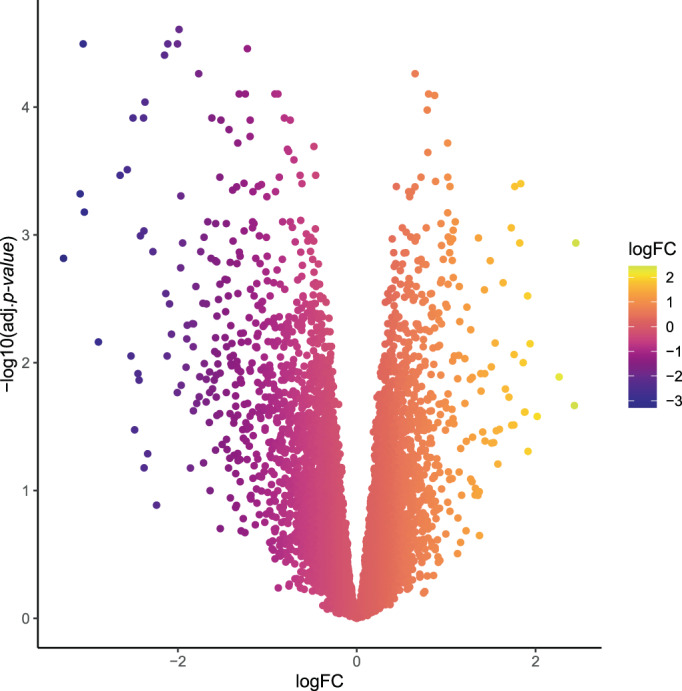
Volcano plots of DEGs. Ggplot2 package in R74 was used to visualize DEGs discovered via meta‐analysis. Cut‐off for *p*‐value was considered .05. It is seen that 30 genes were revealed as common DEGs with a change greater than two folds in chemo‐resistant relative to chemo‐sensitive group. Data are provided as log2 fold change. Ggplot2 R package version 3.3.363 was utilized to create the plots.

**FIGURE 5:**
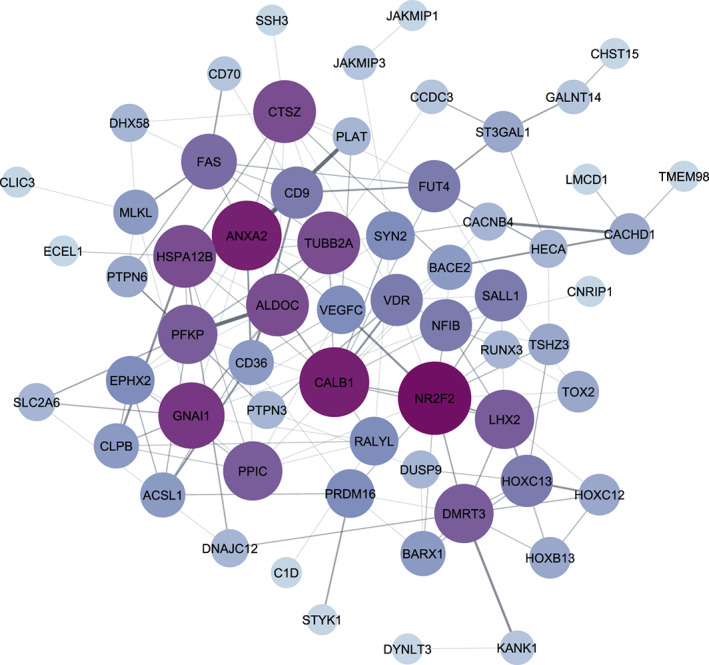
Interaction between genes that are up‐regulated in ovarian cancer cells, cisplatin resistance cell line, A2780CP, relative to cisplatin‐sensitive cell line, A2780S, according to DEGs. Darker color and bigger circles represents higher degree.

**FIGURE 6:**
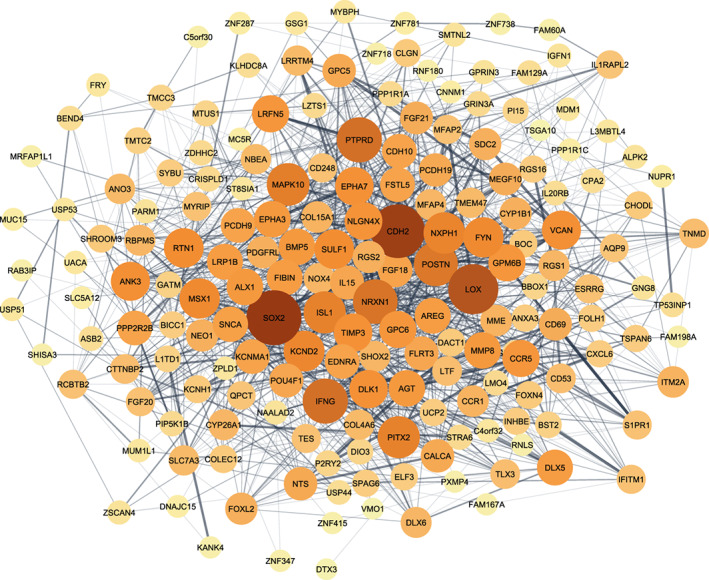
Interaction between genes that are down‐regulated in ovarian cancer cells, cisplatin resistance cell line, A2780CP, relative to cisplatin‐sensitive cell line, A2780S according to DEGs. Darker color and bigger circles represents higher degree.

When all data sets were normalized, integration analysis was conducted in two stages on (1) arrays from OC cell lines (34 arrays) and (2) all normalized arrays from samples taken from patients (23 arrays). Employing the LIMMA method and taking cutoff criterion of adj *p*‐value < .05 led to the identification of 261 DEGs in chemo‐resistant group in comparison with sensitive group for the combination of cell line samples (Figure 4). Figures 5 and 6 demonstrate the interactions between genes with significant up‐ or down‐regulation levels in the first meta‐analysis. Analysis of patients' samples showed that none of the discovered DEGs was statistically significant, thus they were removed from further steps.

Thirty genes out of the 261 DEGs in cell lines with the most significant up‐ or down‐regulation levels (logFC > 2 or log FC < −2) were suggested for further analyses. These genes include *PITX2*, *FIBIN*, *LRRTM4*, *ANO3*, *SNCA*, *FOLH1*, *KANK4*, *FGF18*, *POSTN*, *KANK1*, *GALNT14*, *EPHA7*, *ECEL1*, and *EPHX2* (Figures 5 and 6). This means that these 30 DEGs, consisting of four up‐regulated and 26 down‐regulated genes in cisplatin‐resistant relative to cisplatin‐sensitive OC group play potential roles in drug resistance development in OC. Of these DEGs, *PITX2* gene was found to have the most significant deregulation level with a notable decrease in expression in the chemo‐resistant samples (with logFC, −3.27 and *p*‐value =.0015255). On the contrary, the *KANK1* gene was found to have the most significant increase in the expression (with logFC, 2.45 and *p*‐value = .0011569).

### 3.4 Functional gene enrichment analysis

**FIGURE 7:**
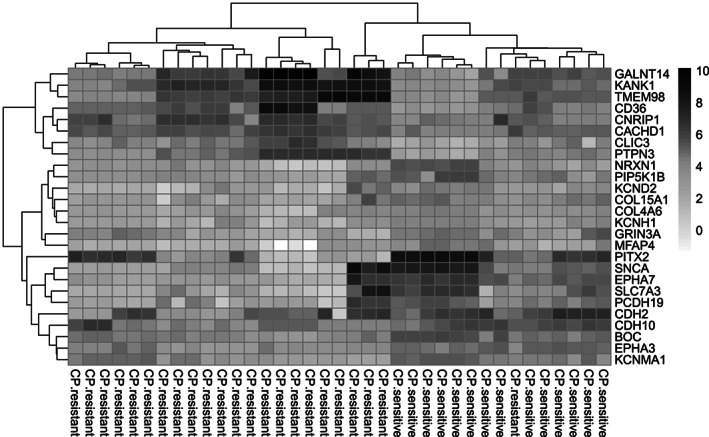
Heatmap of Log fold variations for DEGs with most significant regulation level. Heatmap indicates the normalized relative expression values of DEGs with most significant regulation level involved in drug resistance between cisplatin‐resistant and cisplatin‐sensitive ovarian samples. Each column indicates ovarian samples. All the samples were separated into two clusters according to their characteristics (resistant and sensitive to chemotherapy). Higher gene expression based on microarray data shows with darker color.

Biological functions of DEGs in cisplatin resistance development in OC were revealed through functional gene enrichment analysis using the Enrichr for top up‐ and down‐regulated genes. Enrichr presents GO enrichment comprising categories of molecular function (MF), biological process (BP) and cellular component (CC). Further web‐based pathway analysis was conducted for mapping genes to pathways generated by Kyoto Encyclopedia of Genes and Genomes (KEGG) and Reactome online resources.^14^, ^15^ Our results did not identify any significant signaling pathway related to cisplatin resistance in OC (*p* ≥ .05). In the following, the human protein atlas and GeneCards were used to clarify the biological functions of DEGs. It was revealed that several top up‐regulated genes such as *KANK1*, *GALNT14*, *TMEM98*, and *PTPN3* and top down‐regulated genes such as *PITX2*, *SNCA*, and *EPHA7* play key roles in cancer progression and chemotherapy response. A comprehensive assessment of DEGs revealed significant changes in the expression of several ion transporters such as KCa1.1 (KCNMA1), Kv4 (KCND2), Kv10.1 (KCNH1), GRIN3A, SLC7A3, and CLIC3 and ion transporter regulators such as NRXN1, CNRIP1, and CACHD1. Moreover, the expression of some genes involved in cell adhesion including *MFAP4*, *PCDH19*, *CDH2*, *CDH10*, *NRXN1*, *COL4A6*, *COL15A1*, *PIP5K1B*, *EPHA3*, *BOC*, and *CD36* showed significant changes during cisplatin resistance in OC. To better understand, a heatmap graph of the above‐mentioned genes based on Log Fold change values available in obtained DEGs was depicted (Figure 7).

## 4 DISCUSSION

This study was conducted to investigate deregulated genes and signaling pathways involved in cisplatin resistance development in OC. A total of 261 DEGs were identified. Given that the most significant up‐ and down‐regulated genes in our meta‐analysis have potential roles in drug resistance development, we tried to find the biological roles of 30 annotated genes, the most significant up‐ and down genes, and their relation with drug resistance emergence. Regarding the fact that several molecular mechanisms such as decreased drug uptake and increased drug efflux are involved in cancer drug resistance,^7^ we focused on membrane ion channels, pumps, and transmembrane proteins as well as their regulators in obtained DEGs. Astonishingly, among top up‐regulated genes in the current meta‐analysis, the role of *GALNT14* and *TMEM98* genes in the development of drug resistance have been confirmed previously which was in line with our results. GALNT14 is a mediator of O‐glycosylation whose relation with different tumors are known.^16^ Overexpression of *GALNT14* is correlated with poor prognosis and disease progression in high‐grade serous OC.^17^ GALNT14 regulates the stability of P‐gp, an efflux pump in the cell membrane which leads to an increase in the efflux of anticancer drugs outside the cell and the development of multidrug resistance in breast cancer.^18^ In addition, TMEM98 is a member of single‐pass transmembrane proteins of the endoplasmic reticulum that shows dysregulation in various cancers. The contribution of this family members to drug resistance has been reported in different cancers. For example, the hypomethylation of the promoter region of *TMEM88* gene in OC leads to an increase in its protein expression and development of cisplatin‐resistance.^19^

Besides, a comprehensive investigation of DEGs obtained in this study revealed changes in the expression of some ionic channels KCal.1, Kv4, Kv10.1, GRIN3A, SLC7A3, and CLIC3 as well as their regulating molecules including NRXN1, CNRIP1, and CACHD1. Ionic channels have recently been revealed as key players in drug resistance. The participation of KCal.1 in drug resistance has been established. The expression of this gene is inversely related with drug resistance, which confirms the results of our study. The knockdown of *KCal.1* using the specific siRNA leads to increased sensitivity to cisplatin in drug‐resistant ovarian cell line.^20^ A decrease in the expression of Kv4 was revealed in the results of our study. Although the participation of this channel in drug resistance has not been explored, the dysregulation of other members of this family in developing drug resistance has been established in different studies. For example, the suppression of kv1.5 via potassium channel blockers or specialized siRNA leads to an increase in doxorubicin resistance in gastric cancer.^21^ In addition, Kv1.1 and Kv1.3 lead to the sensitivity of tumor cells and induction of cell death by cisplatin.^22^ An increase in *CACNB4* expression encoding the β subunit of voltage‐gated calcium channels was confirmed in our study. Different studies have established the participation of T‐type and L‐type Ca2+ channels in drug resistance.^23^ Since ionic channels in theory can be easily modulated, they serve as attractive therapeutic targets and pave the way for the development of personalized therapies aimed at stopping or slowing drug resistance process through ionic channel modifications.^24^

Contribution of the PI3K/AKT/mTOR pathway to chemotherapy resistance has been confirmed in various cancers.^25^, ^26^ For example, Ng et al. (2014) observed that the *TMEM98* gene played a critical role in the emergence and development of chemotherapy‐resistance in hepatocellular carcinoma through activating AKT signaling pathway and deactivating p53.^27^ Furthermore, among top‐down‐regulated genes, the role of *PITX2*, *SNCA*, *EPHA7* genes in the development of drug resistance have been confirmed previously which was in line with our results. Based on the results of the current report, the *PITX2* gene has the most significant decrease in the expression. PITX2 plays diverse and complex roles in carcinogenesis. The reduced expression of this gene due to hypermethylation is established in patients with breast, prostate, and clone cancers.^28^ However, in some tumors, this gene plays an oncogenic role and induces cell growth and proliferation.^29^ Lee et al. (2019) established that this gene increases drug resistance in clone and kidney cancers.^30^ Decreased expression of *PITX2* leads to a decrease in B55α production and subsequently prevents the inhibition of AKT, and it may lead to the emergence of cisplatin resistance in OC through the activation of the PI3K/AKT pathway.^28^ Moreover, SNCA (α‐Synuclein) is involved the development of various tumors. The reduced expression of this gene has been established in breast, bladder, kidney, lung, ovarian, brain, and CNS cancers. By reducing the activity of PI3K/AKT, this gene inhibits the proliferation of lung adenocarcinoma cells.^31^ *EPHA7* is a member of the protein‐tyrosine kinase family and plays a dual part of an oncogene or tumor suppressor in intercellular signal transduction and the regulation of cell proliferation and differentiation as well as resistance to chemotherapy drugs.^32^, ^33^ Besides, down‐regulation of *SNCA* and *EPHA7* genes has been associated with multidrug resistance in OC.^34^ The PI3K/AKT signaling pathway is the most common changed signaling pathway in OC and dysregulation of this pathway has been identified in 70% of OC patients.^35^ The activation of this pathway is associated with aggressive phenotype, poor prognosis, and drug resistance in OC.^36^ The level of phospho‐Akt and its kinase activity is significantly higher in OC cells resistant to cisplatin than those sensitive to it. A decrease in *PTEN* and increase in *PIK3CA* lead to the development of cisplatin resistance in OC OVCAR‐3/CDDP cell line. Through inhibiting the release of cytochrome c and the transcription of pro‐apoptotic *Bad* and *Bax* genes, Akt inhibits apoptosis and develops cisplatin resistance in cancer.^37^ Using the specialized inhibitors of this pathway is an efficient and safe strategy to overcome drug resistance without affecting the normal cell performance in the cancer tissue.^38^ Therefore, the use of the specialized suppressors of the genes of this pathway and related proteins, in combination with chemotherapy drugs is proposed as a highly efficient therapeutic strategy to combat drug resistance.

Furthermore, the expression of some genes involved in cell adhesion was found to be dysregulated in the current meta‐analysis. Cell adhesion‐mediated drug resistance inhibits chemotherapy‐induced apoptosis in OC and plays an important role in drug resistance in this cancer.^39^ The findings revealed expression variation in several proteins participating in cell adhesion including MFAP4, PCDH19, CDH2, CDH10, NRXN1, COL4A6, COL15A1, PIP5K1B, EPHA3, BOC, and CD36. Downregulation of *COL4A6* and *EPHA3* genes was found in DEGs obtained in our study. COL4A6 is involved in ECM‐receptor interaction. The expression of this gene decreased in drug‐resistant cell lines and contributed to the development of drug resistance by preventing the penetration of the drug into the cancerous tissue.^40^ The knockdown of *EPHA3* gene led to the appearance of the resistant SCLC phenotype due to apoptosis reduction and induced G2/M phase arrest, and the re‐expression of this gene led to the inversion of the resistant phenotype in the above cells.^41^ Preclinical studies show that targeting molecules participating in cellular attachments including integrins, adapter proteins, and associated kinases can be proposed as a promising strategy to increase the sensitivity of cancer cells to chemotherapy and radiotherapy as well as development of synthetic lethal approaches by multi‐targeting in combination with radio‐chemotherapy in future.^42^

Cumulatively, the results of this study were obtained via investigating high‐throughput studies using in silico tools to predict molecular mechanisms of cisplatin resistance, therefore, using the results, researchers can develop hypotheses for future experimental studies in the area of drug resistance and targeted therapy. The limitations of this study are as follow: absence of independent in vitro and/or in vivo validations, dependency on low‐resolution microarray data, and few numbers of assessed data sets.

## 5 CONCLUSION

In summary, the identification of genes related to drug resistance using bioinformatics tools can be suggested as an important approach for the detection of therapeutic targets for future experimental and preclinical studies aimed at overcoming drug resistance. In this study, we identified some of important mechanisms of drug resistance such as change in drug uptake and increased drug efflux via dysregulation of pumps, ion‐channels and transporters, dysregulation of the PI3K/AKT signaling pathway via upstream up‐regulators and miss‐expression of genes involved in cellular attachments. These mechanisms are proposed as important candidates for future in vitro/ in vivo and targeted therapies.

## AUTHOR CONTRIBUTIONS

**Somayeh Hashemi Sheikhshabani:** Conceptualization (equal); investigation (equal); methodology (equal); validation (equal); visualization (equal); writing – original draft (equal). **Zeinab Amini‐farsani:** Conceptualization (equal); data curation (equal); investigation (equal); methodology (equal); writing – original draft (equal). **Nesa Kazemifard:** Formal analysis (equal); methodology (equal); software (equal); validation (equal); visualization (equal). **Parastoo Modarres:** Conceptualization (equal); investigation (equal); methodology (equal); resources (equal); software (equal). **Zahra Amini‐farsani:** Formal analysis (equal); methodology (equal); resources (equal); software (equal); validation (equal). **Mir Davood Omrani:** Conceptualization (equal); investigation (equal); writing – review and editing (equal). **Soudeh Ghafouri‐Fard:** Conceptualization (equal); project administration (equal); supervision (equal); validation (equal); writing – review and editing (equal).

## CONFLICT OF INTEREST STATEMENT

The authors have stated explicitly that there are no conflicts of interest in connection with this article.

## Data Availability

The sequencing data used in this work are available in the Gene Expression Omnibus (GEO) accession numbers GSE73935, GSE51683, GSE23553, GSE33482, GSE15372, and GSE131978.
